# Public attitudes and health law in conflict: somatic vs. mental care, role of next of kin, and the right to refuse treatment and information

**DOI:** 10.1186/s12913-020-05990-0

**Published:** 2021-01-04

**Authors:** David Wikstøl, Reidar Pedersen, Morten Magelssen

**Affiliations:** Centre for Medical Ethics, Institute of Health and Society, University of Oslo, Pb. 1130 Blindern, N-0318 Oslo, Norway

**Keywords:** Clinical ethics, Health law, Next of kin, Patient autonomy, Survey

## Abstract

**Background:**

Norwegian law and regulations regarding patient autonomy and the use of coercion are in conflict with the Convention on the Rights of Persons with Disabilities (CRPD) and the Oviedo Convention on several points. A new law concerning the use of coercion in Norwegian health services has been proposed. In this study we wanted to investigate the attitudes of the Norwegian lay populace with regards to some of these points of conflict.

**Methods:**

An electronic questionnaire with 9 propositions about patient autonomy, the use of coercion, the role of next of kin, and equality of rights and regulations across somatic and mental health care was completed by 1617 Norwegian adults (response rate 8.5%).

**Results:**

A majority of respondents support the patient’s right to refuse treatment and information in serious illness, that previously expressed treatment preferences should be respected, that next of kin’s right to information and authority in clinical decision-making should be strengthened, and that this kind of legal regulations should be equal across somatic and mental health care.

**Conclusions:**

The findings in this study suggest that the opinions of the Norwegian lay populace are in conflict with the national law on several points relating to patient autonomy, the role of next of kin and use of coercive measures, and different legal regulation of somatic vs. mental health care. The study suggests that the populace is more in line with the CRPD, which supports equal rights across somatic and mental health care, and the Oviedo Convention, which does not allow for the same degree of strong paternalism regarding coercive measures as the current Norwegian law. This can be taken to support the recently proposed legislation on the use and limitation of coercion in Norwegian health services.

**Supplementary Information:**

The online version contains supplementary material available at 10.1186/s12913-020-05990-0.

## Background

Respect for patient autonomy is a central principle in health care ethics [1]. According to this principle the patient has the right to refuse treatment and information, even in cases where treatment or information is strongly advised by health care professionals and refusing has a high risk of leading to serious health deterioration or even death. Health care professionals, on the other hand, have a duty to give the best possible treatment and care necessary to safeguard the patient’s best interests. This duty is by and large taken care of through the principle of beneficence, but also through the principle of non-maleficence. Sometimes, a patient may harm others due to illness, e.g. if the patient is aggressive or violent because of mental illness or dementia. In such cases, the principle of justice comes into play, entailing that all humans should have equal rights to freedom and to protection from harm from others.

When these principles come into conflict it is generally recognized that in some cases, it is right for the health care professional to limit the patient’s autonomy for the best interest of the patient or to protect other interests. This may lead to the use of coercion, which is relatively common both in mental and somatic health care [2, 3]. To balance these principles, health care practice is regulated by national health laws. Using coercion in health care without a legal basis is generally regarded as illegal and also a violation of basic human rights.

In Norway these principles are regulated by a set of different and often complicated laws, which in many respects are difficult to interpret and in some cases can leave the clinician uncertain of what he has the right to do [4]. What is clear, however, is that cases of mental illness are not regulated by the same set of laws as somatic illness, with the result that the mental health care patient does not have the same rights to refuse treatment as the somatic health care patient (somatic health care meaning all health care services except mental health care services, e.g. internal medicine, surgery, neurology, and most of the care provided in nursing homes). These differences are challenged by the Convention on the Rights of Persons with Disabilities (CRPD). There are many interpretations of the CRPD, but one widely shared interpretation is that differences in patients’ rights based upon the dichotomy somatic vs. mental illness put too much emphasis on the type of disorder and thus risk discriminating patients with certain types of disorders. In line with this, many have argued for the need of joint or fusion laws applicable to all persons receiving health care and a focus on the patients’ ability or competence to consent rather than the diagnosis or type of disorder in laws regulating patients’ rights and the use of coercion [5–7]. Another argument for fusion laws is that it may be difficult to draw a clear line between somatic and mental illness, for example with cognitive impairment, eating disorders, and delirium due to somatic illness. To ground the right to refuse health care services in fuzzy concepts increases the risk of unfounded variations in the use of coercion and haphazard discrimination.

To reduce potentials for discrimination and paternalism in mental health care, lack of competence to consent has recently been included as a condition for the use of coercion in Norwegian mental health care legislation. In somatic health care, competence to consent has been a criterion since 2009. However, the recent change has created another type of asymmetry between mental and somatic health care: in mental health care, coercion can only be applied if a patient lacks competence to consent, *and* if the patient’s mental illness is *serious*, i.e. a psychotic disorder or equally severe disorder. This requirement of severity of the illness does not apply to patients with somatic illness. Thus, incompetent patients with a moderate illness refusing treatment can only be given help if the illness is somatic, and not if it is a moderate mental illness [8]. This may make it impossible to perform “weak” paternalism, e.g. to help patients with moderate mental health problems lacking competence to consent and refusing treatment due to cognitive impairment (which is not defined as a serious mental disorder in Norway). This also applies if the patient has previously expressed wishes indicating that he or she would have wanted treatment, e.g. for depression or anxiety to raise quality of life, under these circumstances.

Another asymmetry in the regulations regarding somatic and mental illness is that there is no opening for the use of coercion in somatic health care *to protect the interests of others*, except in cases where the duty of emergency health care applies (see below) [8]. That is, the coercion regulations in somatic health care in general follow the so-called “best interest standard” where the coercive measures have to be in the interest *of the patient*. In mental health care, on the other hand, compulsory care may be provided if the patient suffers from a serious mental disorder and there is “an obvious and serious risk to his or her own life *or the life or health of others*” (our emphasis). This is possible also when the patient is competent [9].

Another point of concern is that the “duty of emergency health care” in Norwegian health law allows for more paternalism than in other Western countries and in the Oviedo Convention [10, 11]. This duty cannot be used for involuntary admissions in mental health care, thus it is mostly used in somatic health care. When this duty applies, the use of coercion is not only allowed, but also required, and the duty also applies to patients competent to consent (i.e. “strong” paternalism) and even when there is enough time and possibilities for adequate clinical communication. The conditions necessary for this duty to apply are defined vaguely (“that the health care is urgently necessary”), and this opens up for “strong” paternalism in many circumstances [4, 12]. Furthermore, it may be used to protect the interest of others (see above).

In Norwegian law, the patient’s rights to refuse *treatment* differ from the rights to refuse health-related *information*. Whereas patients as a main rule (with the exceptions described above) can refuse treatment, allowing the patient to refuse information presupposes that not giving the information is “acutely necessary to prevent risk of death or serious injury for the patient” or “clearly inadvisable […] out of consideration for persons close to the patient.” [13]. The health-related information in question is the information necessary for the patient to gain insight into their own health condition, what the treatment amounts to, and possible effects, risks and side effects. The strict criteria for the possibility to refuse information creates somewhat of a discrepancy in the law in regards to patient autonomy. The law by and large respects patient autonomy when it comes to refusing treatment, but in most cases it would require full disclosure of health related information *even if* the patient were to prefer not to receive the information or would rather leave it to the professionals to decide. This is sometimes called a “waiver” and is allowed for in many other countries’ health care legislation to avoid “information paternalism”. One could argue that such a waiver undermines the possibility of an informed consent and thus also conflicts with respecting the patient’s autonomy. However, it could also be argued that the right not to know, and to allow the patient to hand over more of the decision-making authority to the responsible clinician or another representative, e.g. based on trust, is also a way to respect the patient’s autonomy.

Decision-making authority regarding treatment and access to health-related information for the patient’s next of kin is also regulated by Norwegian law. In questions of whether or not to start treatment for patients without competence to consent, the next of kin’s only *legal* role is to make known what the patient most likely would have wanted. In cases in which the patient’s earlier expressed preferences are known, these preferences are to be respected, though this is not clearly regulated by the law [14]. The responsibility to decide on treatment when the patient lacks competence, lies solely with the responsible health care provider. When it comes to health-related information, next of kin have in general no right to receive health-related information about the patient without the patient’s own explicit consent. This is the case even in cases where the next of kin are acting as the patient’s most important, yet informal, caregivers and the information is important for them to provide good care. By comparison, sharing information with other health care professionals providing care to the patient does not require an explicit consent when the information is important to provide good care. Here, it is enough that the patient receive general information about information sharing routines and do not make an unprompted refusal (“opt out” rather than explicit informed consent). However, if the patient lacks competence to consent, then the *closest* next of kin has the right to participate in clinical decision making as described above, and thus has a right to relevant information. Thus, an important exception to the duty of confidentiality is towards the closest next of kin when the patient lacks competence to consent [15].

### Aim and contribution

The aim of this study is to inquire into whether the opinions of the Norwegian populace is in concordance with, or diverges from, the national laws regulating the patient’s rights to refuse treatment and information, respect for earlier expressed preferences, the role of next of kin in regards to information and decision making, and the differences between laws regulating somatic and mental illness.

Although the starting point for this article is the Norwegian health law, many countries have similar legislation, for example separate legislation on the use of coercion in mental health care. Furthermore, while the emphasis on patient autonomy is in general increasing worldwide, and a few countries have already adopted fusion laws [16], many countries are now discussing how to best secure patient autonomy and involvement of relatives and how to best comply with the CRPD and the Oviedo Convention.

Information about the opinions of the populace is of general interest because they provide information about the legitimacy, or lack thereof, for the legal regulations, and may also indicate that parts of the law should be amended. The results are also of special relevance for countries considering changing their health laws on coercion or on involvement of relatives, e.g. due to the CRPD or to support informal caregivers. One common strategy here is to make lack of competence to consent a key criterion for the use of coercion (often together with other criteria).

In Norway the topic is of particular interest because of the newly proposed law on use of coercion in Norwegian health services, in which coercion in both somatic and mental health care services is suggested to be regulated by the same law [17].

## Methods

This study was one of four sub-studies in the 2017 NOBAS (Norwegian Bioethics Attitude Survey) study. In February 2017, the commercial research firm Respons Analyse invited members of their nationally representative web panel via email to complete the NOBAS online questionnaire, which they were told would assess attitudes towards ethical issues. A total of 18,976 respondents were invited by email, and the online questionnaire remained open until the target number of responses (> 1500; a number decided upon in light of power calculations for one of the other substudies, and deemed sufficient also for the present substudy) had been reached. Thus, non-responders could either have declined to participate or not have considered the invitation before the questionnaire was closed. One thousand six hundred seventeen respondents completed the survey; divided with the total number of invitees this gives a response rate of 8.5%. The answers were weighted so that the answers from underrepresented groups in the study were given more weight in the analyses (for further information about weighting see Table A1 in Additional file 1).

This part of the survey consisted of nine questions. All of the respondents were presented with the same questions, but were randomized to receive one of two clinical vignettes. Vignette 1 described a patient with an acute somatic illness (acute myocardial infarction): “A 60-year-old woman suffers a myocardial infarction and is admitted to the hospital. In spite of severe pain, the woman is able to understand the situation and make her own choices. The doctor recommends treatment in the form of opening the blocked coronary artery by way of a “stent” inserted by a catheter. Without this treatment, there is an increased risk of serious damage to the heart and in the worst case death.”

Vignette 2 described a patient with an acute mental illness (acute psychosis): “A 60-year-old woman develops a serious mental illness (psychosis) and is admitted to the hospital. In spite of her mental illness, she is able to understand the situation and make her own decisions. The doctor recommends treatment in the form of medication (“antipsychotic drugs”). Without this treatment, there is an increased risk that the illness becomes protracted.”

Apart from presenting two different diseases we attempted to make the two vignettes as similar as possible. The first five questions were related to the vignette and concerned the patient’s opportunity to refuse treatment or information, and the role of next of kin and the doctor in making the decision:

- Q1. The patient should have the right to refuse treatment.
- Q2. Given that the patient wants the treatment but does not want information about the illness and the risks/side effects associated with the treatment, then this should be respected.
- We then prefaced the next three questions with this paragraph: “Now imagine that the patient is not capable of understanding the situation or make her own decisions, and that her closest next of kin are her husband and daughter. Take a stand towards the following propositions:”
- Q3. If the patient on an earlier occasion, while she was capable of making her own decisions, had clearly expressed her preferences as not to want treatment in case of serious illness, then this should be respected.
- Q4. When the patient is not capable of making her own decisions, next of kin should have the authority to decide in questions of treatment, so long as the patient herself, on an earlier occasion, has expressed the preference that her next of kin should have this role.
- Q5. When the patient is not capable of making her own decisions, the doctor should have the authority to decide in questions of treatment, and not the next of kin.

The four last questions did not refer to the vignette, and here responses were analysed for the total set of respondents together. They concerned the next of kin’s rights and access to information, the use of coercion for consideration towards others than the patient, and whether somatic and mental health care services should have the same regulations in regard to patient autonomy, the use of coercion and collaboration with next of kin. These questions were prefaced with this introduction: “As it stands today, next of kin have no independent right to information about a patient with competency of consent. This is also the case in situations where the patient is dependent on care from next of kin. The health care professional caring for the patient, on the other hand, is allowed relevant information without consulting the patient.”

- Q6. If a patient is dependent on care from next of kin, then these next of kin should have the same right as health care professionals to information about the patient which can make it easier to care for the patient.
- Q7. If a nursing home patient verbally abuses other patients or is acting in a threatening manner, then the health care professional should have the possibility to use coercion against the patient to shield the other patients from this patient.

The final two questions were introduced thus: “As it stands today, the laws regulating the use of coercion and patient autonomy are different between mental health care and somatic (bodily) health care.”

- Q8. Patients who are capable of making their own decisions should have the same right to refuse treatment, independent of whether they are suffering from a mental or bodily (somatic) illness.
- Q9. The criteria for the use of coercion and the regulations regarding the role of next of kin should be the same independent of whether the patient is suffering from a mental or bodily (somatic) illness.

All the questions were posed as propositions to which the respondents had to answer to which extent they agreed. The alternatives were “completely disagree”, “somewhat disagree”, “neither disagree or agree”, “somewhat agree” and “completely agree”. Every question had to be answered for the answer to be registered. The questions and vignettes were presented in Norwegian.

The survey was validated by testing the questionnaire on four laypersons who gave detailed feedback, and by discussions among professional experts. The electronic questionnaire was pilot tested.

Statistical analyses were performed in IBM SPSS Statistics 26. Likert scores were calculated on a 5-point scale with “completely disagree” = 1 and “completely agree” = 5. The differences between the groups of respondents were analysed by the Wilcoxon–Mann–Whitney test and statistical significance defined as *p* < .05.

## Results

A majority of the respondents agreed (completely or somewhat) that the competent patient should have the right to refuse treatment (Q1; Table 1). However, there were far more respondents who agreed that the patient should have the right to refuse treatment in the case of myocardial infarction (71%) than in the case of psychosis (55%; *p* < 0.001) (Q1). A majority agreed that the patient’s preference not to receive information (Q2) and earlier expressed preferences not to receive treatment (Q3), should be respected, and here there were no group differences. There were more respondents supporting the proposition that *the closest next of kin* should have a deciding role when the patient lacked competence to consent, than there were respondents supporting the proposition that *the doctor* should have decision-making authority (Q4–5). Here, support for the next of kin’s decisional authority was stronger in the psychosis than in the myocardial infarction case.

**Table 1:** The respondents’ views on the patient’s right to refuse treatment and information, and the role of doctors and the closest next of kin in decision-making. Percentage. *M.I*. myocardial infarction. *P*-value indicating differences between the M.I. and psychosis groups. *ns* non significant

		Completely/ somewhat disagree	Neither disagree or agree	Completely/ somewhat agree	Mean Likert score	*p*-value
**Q1 (right to refuse treatment)**	M.I.	19	10	71	3.87	< 0.001
	Psychosis	31	13	55	3.45	
**Q2 (right to refuse information)**	M.I.	26	15	60	3.55	.26 (ns)
	Psychosis	22	16	63	3.64	
**Q3 (respect for earlier preferences)**	M.I.	11	10	79	4.13	.067 (ns)
	Psychosis	15	9	76	4.02	
**Q4 (role of next of kin in decision making)**	M.I.	10	9	81	4.17	.026
	Psychosis	5	9	86	4.31	
**Q5 (role of the doctor in decision making)**	M.I.	41	20	39	2.98	.249 (ns)
	Psychosis	41	20	38	2.93	

A clear majority of the respondents agreed (completely or somewhat) that next of kin should have the right to information about the patient when the patient is dependent on care from next of kin (Q6; Table 2), and that the use of coercion against nursing home patients should be allowed in order to secure the safety of other patients (Q7). A majority also agreed that patients with competence to consent should have the same right to refuse treatment independently of whether the illness is mental or somatic (Q8). A majority did also agree that the criteria for the use of coercion and regulations regarding the role of next of kin should be the same for mental health care and somatic health care (Q9).

**Table 2:** The respondents’ views on next of kin’s right to information, use of coercion and equal regulations in mental and somatic health care. N (%)

	Completely disagree	Somewhat disagree	Neither agree nor disagree	Somewhat agree	Completely agree	Mean Likert score
**Q6 (next of kin’s right to information)**	31 (1.9)	90 (5.5)	117 (7.3)	452 (28.0)	927 (57.4)	4.33
**Q7 (coercion for the safety of others)**	100 (6.2)	143 (8.9)	246 (15.2)	620 (38.4)	507 (31.4)	3.80
**Q8 (equal right to refuse treatment)**	94 (5.8)	311 (19.2)	280 (17.3)	463 (28.6)	469 (29.0)	3.56
**Q9 (equal regulations)**	89 (5.5)	284 (17.5)	353 (21.8)	453 (28.0)	439 (27.1)	3.54

## Discussion

In this section, we will offer some interpretations of the respondents’ answers to each question and make clear on which points the results suggest that the opinion of the Norwegian lay populace is either in conflict or in line with national law, the CRPD and the Oviedo Convention.

Q1 shows a majority of the respondents agreeing (completely or somewhat) that the patient, in both cases presented as having competence to consent, should have the right to refuse treatment in serious cases like acute myocardial infarction and acute psychosis. As it stands today the case of acute myocardial infarction would fall under the duty of emergency health care and the patient would *not* have the right to refuse treatment, which would be in conflict with the opinion of the majority of respondents in this survey. In the case of acute psychosis there is not described a situation of great danger for the patient’s own life or the life and health of others, and the case therefore does not fulfil the requirements for compulsory mental health care. This is in line with the opinion expressed by the majority of respondents. There was greater support for the patient’s right to refuse treatment in the case of serious somatic illness than serious mental illness. This is somewhat counterintuitive in regards to the particular cases since the case of myocardial infarction has a higher probability of imminent death than that of psychosis, and has a higher probability of regaining health if given treatment. One possible explanation of the difference is that, even though the mental health care patient is presented as being “able to understand the situation and make her own decisions”, laypersons can nonetheless have a strong association between mental illness, psychosis specifically, and not having competence to consent. The respondents’ prejudices may overshadow the information given about the patient’s competence to consent. Dependent on the impact of this suggested effect, the responses to Q1 may suggest some support for treating cases of serious somatic illness and serious mental illness differently, or suggest the existence of prejudices against the possibility that persons with psychosis may be competent to consent.

A majority of the respondents supports the patient’s rights to refuse information (Q2), with only minor differences between the somatic and mental illness cases. This is in contrast with the current national law. As it stands today, the strict criteria for the possibility to refuse information would not be met in either of these situations, and the health care professionals would have a duty to give information in spite of the patient’s preference. Here the intuitions of the respondents seem to favor the principle of patient autonomy and could therefore be taken as support for a change in legislation.

Q3 shows that a majority of the respondents supports the patient’s rights to refuse treatment based on earlier expressed preferences, both for somatic and mental illness, if the patient presently lacks competence to consent. This is supported in national law, both for somatic and mental health care, but is not clearly regulated.

Q4 and Q5 show the respondents giving far greater support for the proposition that *the closest next of kin* should have decision-making authority regarding treatment, if in accordance with the patient’s earlier expressed preference, than for the proposition that *the doctor* should have the deciding role regarding treatment. One possible explanation of this difference is that Q4 was posed before Q5 and that without being aware of other possibilities, or the implications the proposition, Q4 immediately seemed reasonable to the respondents. Then being presented with Q5 they had already committed to the reasonableness of Q4, and then saw Q5 as less reasonable. This could be an instance of the effects of question order widely recognized in survey psychology [18]. The rise in the percentage of respondents who neither agreed nor disagreed supports this hypothesis by indicating a rise in uncertainty presented with an option of a contrary perspective. This effect could be reduced or avoided if we had randomized the order of Q4 and Q5 in the study design. Another possibility is, of course, that the respondents genuinely tend to think that the next of kin should in some cases have the deciding role and not the doctor. If so, then this is significant since it suggests that opinions may go against current national law and practice which clearly assign ultimate decision-making authority to the doctor. Further inquiry is needed to settle this.

The respondents’ answers to Q1-Q5 suggest that the majority of the Norwegian lay populace may put greater emphasis on patient autonomy than the current Norwegian health legislation, and align very well with the provisions in the Oviedo convention [10].

Q6 shows a clear majority of the respondents agreeing with the proposition that next of kin should have the right to information about the patient when the patient is dependent on care from next of kin. One could argue that the isolated focus on care and the role of next of kin as carer would prompt the respondents to agree with the proposition. Had the respondents also been presented with a situation where a patient preferred to decide what kind of information that should be shared with next of kin, or had reasons for this, it is possible that a significant part of the respondents would have answered otherwise. The response to Q6 is nonetheless interesting because it goes against current law and practice, where the next of kin does not have any right to information, and can only get information with the explicit consent of the patient, given that the patient has competence to consent, which in turn can serve as an obstacle to provide necessary support and information to the next of kin, and thus for the informal care given to the patient.

Q7 shows a clear majority of the respondents agreeing with the proposition that the use of coercion against nursing home patients should be allowed if necessary to secure the safety of other patients. This finding is significant because the use of coercion due to the safety of others is illegal in the current regulation of *somatic* health care, which is the regulations most often used in cases of dementia [19]. If the case were to go under the regulations of *mental* health care, which in some cases take into account the safety of others, it would not allow the use of coercion since dementia is generally not considered a serious mental disease.

Q8 shows that a majority of 58% of the respondents agreed (somewhat or completely) that patients with competence to consent should have the same right to refuse treatment independently of whether the illness is mental or somatic. This is in line with article five of the CRPD, which requires non-discrimination and equality for all persons under the law independent of the type of disorder. Interestingly, the respondents who were presented with the mental illness vignette were, as mentioned in the results, more likely to agree than those who were presented with the somatic vignette. One possible explanation of this difference is that mental illness may be a stronger reminder of possible discrimination than somatic illness or that those presented with the somatic case were not presented with a particular case of a mentally ill patient with competence to consent and may therefore be less likely to see this as a possibility. That a majority of the respondents supports equal regulations can give rise to a paradox in relation to the findings of Q1, where the answers suggested that a significant portion of the respondents supports that the cases should be treated differently. It may be that posing the question in a principled manner, instead of particular cases, elicits other intuitions based on more general views of equal rights. It could also be the case that since key principled information is presented together with Q8 than Q1 it is easier to think principled and avoid bias.

Q9 shows that a majority of 55% of the respondents agreed (somewhat or completely) with the proposition that the criteria for the use of coercion and regulations regarding the role of next of kin should be the same for mental health care and somatic health care (against 23% who disagreed somewhat or completely and 22% who neither agreed nor disagreed). The percentages mirror the ones in Q8 and show that a majority of the respondents supports these forms of equality of rights and regulations between somatic and mental health care.

The results suggest that the opinions of the populace diverge from the current regulations on several points. Whether this should be taken as justification for a change in regulations is dependent on the validity of the results (discussed under Limitations) and the validity of the proposed interpretations. It is also dependent on whether and to what extent popular opinion in general should influence the regulation of health care. The fact that everyone in the population is a potential patient, and therefore is directly concerned, speaks in favor of this. The fact that medicine and ethics is a field of expertise, where the health care professional or ethicist is arguably in the best position to know and evaluate all the relevant factors and implications for such regulations speaks against this view. Ideally, good reasons or arguments alone should decide regulations, and not opinions or intuitions. One could object that reasons, or the reasoning of experts, is always ultimately based on intuitions, but these intuitions would be significantly more basic and better informed than the intuitions of laypeople presented with complex cases that they have not previously reflected on or had experience with. Nevertheless, laws are established as part of a democratic process, and if the opinions of the populace are in concordance with well thought out reasons and principles, they can, and perhaps should, serve as a support for developing laws in accordance with these reasons and principles and secure more democratic regulation of health care.

### Limitations

According to the belief-sampling model of survey response, the attitudes tested by such surveys are seen as “a kind of memory structure that contains existing evaluations, vague impressions, general values, and relevant feelings and beliefs” [18]. Thus, answers based on this “memory structure”, from respondents presumed to be largely unfamiliar with these particular ethical questions, are more “intuitions” than they are considered moral judgments. The results of the survey should be interpreted in this light.

It is also the case that there could be several important factors related to the clinical context which are left out in these simplified thought experiments. There could, for example, be different circumstances relating to the reliability of next of kin’s account of the patient’s past verbal expressions of preferences, potential conflict of interest, financial or otherwise, between the patient and next of kin, or between next of kin. As the cases in this study do not include such factors, the responses can be taken to represent the broad attitudes of the population towards some of the principles involved in these ethical questions not including complicating factors.

The low response rate of 8.5% raises the question of the validity of the findings. With such a low response rate there is a potential for non-response bias, i.e. that there is a significant difference between the people who chose to participate in the study and those who declined, so that the results become less representative of the population in general. There are, however, some reasons to believe that a non-response bias does not affect the results of this study to a significant degree (some of which are also discussed in greater depth in an earlier article from the 2017 NOBAS study [20]).

Most importantly, the randomization of respondents to receive either one of two different vignettes counteracts any sample biases. Randomization will have distributed relevant factors equally among the two groups. There is a high correspondence between the demographic characteristics of the national average and that of the respondents, and the answers from underrepresented groups were given more weight to further increase this correspondence. Furthermore, there is evidence that non-response bias poses less problems in surveys about respondents’ attitudes than those involving respondents’ self-reported activities [21]. Fourthly, the invitation email did not disclose the nature of the topics of the survey. The survey was presented as a «survey on attitudes towards ethical questions», and could therefore lead to respondents with high interest in ethical questions in general to be more likely to participate. Nevertheless, since there is no reason to believe that a higher interest in ethical questions would correlate with a preference for certain views, this would not likely lead to any bias effects on the results (perhaps with the exception of a lower level of indifferent answers) [22]. The survey also consisted of different parts, of which all had to be completed, which further reduces bias towards a particular topic.

The problem of low response rate is a general problem in population survey-research, and detailed analyses indicate that, although a survey has a very low response rate, the survey may nevertheless be representative of the population [21, 22]. There are therefore several reasons why non-response bias does not significantly affect the results of this survey. Thus, even though the results must be taken to contain a certain degree of uncertainty, they can still be seen as good indications of the true opinions of the populace.

## Conclusion

The findings in this study suggest that a majority of the Norwegian lay populace may be in conflict with the national law on several points relating to patient autonomy, the role of next of kin and use of coercive measures, and the differences between the regulations of somatic and mental health care. Contrary to national law the majority of the respondents supported the patient’s right to refuse treatment and health related information in situations of serious somatic or mental illness, given that the patient has competence of consent. The majority of the respondents, also in conflict with current national law, supported that closest next of kin should have decision-making authority regarding treatment and not the doctor, in situations where the patient, currently lacking competence to consent, has previously expressed that next of kin should have this role. The majority of the respondents also supported the right to health related information for close next of kin, when the patient is dependent on care from next of kin, also when the patient has competence to consent. Such a right does not exist today, and this may severely reduce the quality of informal care provided. The majority of the respondents, again against national law, supported that use of coercion against nursing home patients should be allowed to secure the safety of other patients. With regards to the differences in regulations of patient autonomy and the use of coercion between somatic vs. mental health services, the majority of the respondents supported equal rights and regulation.

This study suggests that the populace is more in line with the CRPD, which supports equal rights across somatic and mental health care, and the Oviedo Convention, which does not open for the same degree of strong paternalism regarding coercive measures as the current Norwegian law. This is also in line with, and can be taken to support, the newly proposed law on the use and limitation of coercion in Norwegian health services, in which coercion in somatic and mental health care services is suggested to be regulated by the same rules.

## Supplementary Information

**Additional file 1: Table A1.** Demographic characteristics of respondents.

## Data Availability

The data are available on reasonable request to the corresponding author.

## Abbreviations

CRPD: Convention on the Rights of Persons with Disabilities
M.I.: Myocardial infarction
NOBAS: Norwegian Bioethics Attitude Survey

## References

[1] Beauchamp TL, Childress JF (2019). Principles of biomedical ethics.

[2] Beghi M, Peroni F, Gabola P, Rossetti A, Cornaggia CM (2013). Prevalence and risk factors for the use of restraint in psychiatry: a systematic review. Rivista di Psichiatria.

[3] Kirkevold Ø, Engedal K (2004). Prevalence of patients subjected to constraint in Norwegian nursing homes. Scand J Caring Sci.

[4] Pedersen R, Nordtvedt P (2006). Når kan pasienten nekte helsehjelp?. Kritisk Juss.

[5] Szmukler G, Holloway F (1998). Mental health legislation is now a harmful anachronism. Psychiatr Bull.

[6] Dawson J, Szmukler G (2006). Fusion of mental health and incapacity legislation. Br J Psychiatry.

[7] Szmukler G, Daw R, Callardc F (2014). Mental health law and the UN convention on the rights of persons with disabilities. Int J Law Psychiatry.

[8] Pedersen R, Pedersen R, Nordtvedt P (2017). Aarre TF. Etikk i psykiske helsetjenester.

[9] Lov om etablering og gjennomføring av psykisk helsevern (psykisk helsevernloven), § 3–3, English translation: https://app.uio.no/ub/ujur/oversatte-lover/data/lov-19990702-062-eng.pdf. Accessed 9 Dec 2020.

[10] Convention for the Protection of Human Rights and Dignity of the Human Being with regard to the Application of Biology and Medicine: Convention on Human Rights and Biomedicine. Oviedo: Council of Europe; 1997.

[11] Nys H, Raeymaekers P (2013). Competence assessment and advance directives for people with dementia: ethical and legal aspects. Brussels the King Baudouin Foundation.

[12] Lov om helsepersonell m.v. (helsepersonelloven), § 7, English translation: https://www.regjeringen.no/no/dokumenter/act-of-2-july-1999-no-64-relating-to-hea/id107079/. Accessed 9 Dec 2020.

[13] Lov om pasient- og brukerrettigheter (pasient- og brukerrettighetsloven), § 3–2, English translation: https://app.uio.no/ub/ujur/oversatte-lover/data/lov-19990702-063-eng.pdf. Accessed 9 Dec 2020.

[14] Lov om pasient- og brukerrettigheter (pasient- og brukerrettighetsloven), § 4–6, English translation: https://app.uio.no/ub/ujur/oversatte-lover/data/lov-19990702-063-eng.pdf. Accessed 9 Dec 2020.

[15] Lov om pasient- og brukerrettigheter (pasient- og brukerrettighetsloven), § 3–3, English translation: https://app.uio.no/ub/ujur/oversatte-lover/data/lov-19990702-063-eng.pdf. Accessed 9 Dec 2020.

[16] Sugiura K, Mahomed F, Saxena S, Patel V. An end to coercion: rights and decision-making in mental health care. Bulletin of the World Health Organization. 2020;98:52–8. 10.2471/BLT.19.234906 PMC6933425 31902962

[17] Østenstad BH (2019). NOU 2019: 14 Tvangsbegrensningsloven — Forslag til felles regler om tvang og inngrep uten samtykke i helse- og omsorgstjenesten.

[18] Tourangeau R, Rips LJ, Rasinski K (2000). The psychology of survey response.

[19] Lov om pasient- og brukerrettigheter (pasient- og brukerrettighetsloven), Kapittel 4 A, English translation: https://app.uio.no/ub/ujur/oversatte-lover/data/lov-19990702-063-eng.pdf. Accessed 9 Dec 2020.

[20] Magelssen M, Solberg B, Supphellen M, Haugen G. Attitudes to prenatal screening among Norwegian citizens: liberality, ambivalence and sensitivity. BMC Med Ethics. 2018;19:80. 10.1186/s12910-018-0319-9 PMC6145324 30227857

[21] Hellevik O (2016). Extreme nonresponse and response bias. Qual Quant.

[22] Fosnacht K, Sarraf S, Howe E, Peck LK (2017). How important are high response rates for college surveys?. Rev High Educ.

