# Supplementary material for: Public attitudes and health law in conflict: somatic vs. mental care, role of next of kin, and the right to refuse treatment and information
Source: BMC Health Serv Res. 2021 Jan 4;21:3. doi: 10.1186/s12913-020-05990-0 (PMC7780687; doi:10.1186/s12913-020-05990-0)
Supplement: Supplementary file 1 — Additional file 1: Table A1. Demographic characteristics of respondents. [file 12913_2020_5990_MOESM1_ESM.docx]

**Public attitudes and health law in conflict: somatic vs. mental care, role of next of kin, and the right to refuse treatment and information**

**Additional file 1.docx**

**Table A1.** Demographic characteristics of respondents

| Characteristic | | Unweighted (N, (%)) | Weighted (N, (%)) |
| --- | --- | --- | --- |
| Sex | Female | 811 (50.2) | 804 (49.7) |
|  | Male | 806 (49.8) | 813 (50.3) |
| Age | 18-24 | 110 (6.8) | 193 (11.9) |
|  | 25-34 | 233(14.4) | 282 (17.5) |
|  | 35-44 | 254 (15.7) | 289 (17.9) |
|  | 45-54 | 308 (19.0) | 287 (17.7) |
|  | 55+ | 712 (44.0) | 567 (35.0) |
| Level of education | Primary school | 74 (4.6) | 74 (4.6) |
|  | Upper secondary school | 396 (24.5) | 430 (26.7) |
|  | College/university ≤3 yrs | 460 (28.5) | 443 (27.5) |
|  | College/university >3 yrs | 658 (40.7) | 647 (40.2) |
|  | Unanswered | 18 (1.1) | 15 (0.9) |
| Religious beliefs | Non-religious | 728 (45.0) | 753 (46.7) |
|  | Christian | 710 (43.9) | 685 (42.5) |
|  | Other religions | 26 (1.6) | 26 (1.6) |
|  | Unanswered | 148 (9.2) | 149 (9.2) |
